# Random Forest Models for Accurate Identification of Coordination Environments from X-Ray Absorption Near-Edge Structure

**DOI:** 10.1016/j.patter.2020.100013

**Published:** 2020-04-21

**Authors:** Chen Zheng, Chi Chen, Yiming Chen, Shyue Ping Ong

**Affiliations:** 1 Materials Virtual Lab, Department of NanoEngineering, University of California San Diego, 9500 Gilman Drive, Mail Code 0448, La Jolla, CA 92093-0448, USA

**Keywords:** machine learning, X-ray absorption spectroscopy, random forest, feature importance, XANES, coordination environment, materials

## Abstract

Analyzing coordination environments using X-ray absorption spectroscopy has broad applications in solid-state physics and material chemistry. Here, we show that random forest models trained on 190,000 K-edge X-ray absorption near-edge structure (XANES) spectra can identify the main atomic coordination environment with a high accuracy of 85.4% and all associated coordination environments with a high Jaccard score of 81.8% for 33 cation elements in oxides, significantly outperforming other machine-learning models. In a departure from prior works, the coordination environment is described as a distribution over 25 distinct coordination motifs with coordination numbers ranging from 1 to 12. More importantly, we show that the random forest models can be used to predict coordination environments from experimental K-edge XANES with minimal loss in accuracy. A drop-variable feature importance analysis highlights the key roles that the pre-edge and main-peak regions play in coordination environment identification.

## Highlights

• Random forest models accurately identify atomic coordination environments from XANES
• World's largest X-ray absorption spectra database
• Transferable model performance to experimental spectra
• New insights from interpreting machine-learning models

## The Bigger Picture

The characterization of atomic local environments in a material is important in many physical and chemical fields. Among various techniques, X-ray absorption spectroscopy (XAS) is one of the most widely used methods. However, the analysis of XAS is often qualitative and contrastive, requiring reference spectra from compounds that may not be available. This work introduces a machine-learning (ML)-based approach that directly predicts the atomic environment labels from the X-ray absorption near-edge structure (XANES) by training on a large computed XANES dataset. This data-driven approach shows excellent accuracy exceeding 80% in both computational and experimental tests. The application of ML models to spectroscopy will likely gather considerable interest in the near future, with accelerated or even on-the-fly interpretation of spectra directly from experiments. Such ML-accelerated approaches are expected to bring about a transformative leap in the pace of materials discovery and design.

## Graphical Abstract

**Figure:**
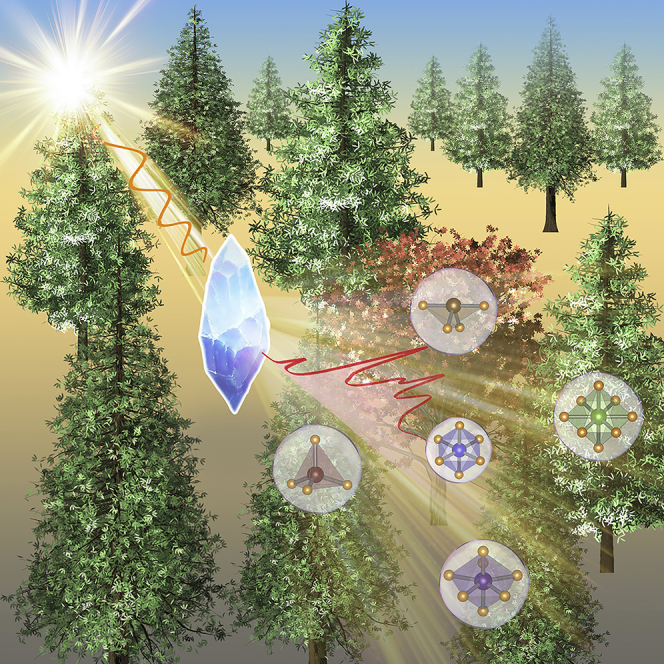

## Teaser

Machine learning (ML) is rapidly changing the landscapes of physical and chemical science. However, material science data are often too small for ML models. In this work, random forest models are trained on the world's largest computed X-ray absorption spectroscopy database that contains 190,000 spectra and are used to accurately predict atomic local environments therein. The models show outstanding accuracy exceeding 80% and are able to extract chemical insights that extend previous knowledge.

## Introduction

X-ray absorption spectroscopy is an important technique for probing the local environments, i.e., atomic coordination symmetries, the number and chemical identities of neighboring atoms and oxidation states, in a material.^1^, ^2^, ^3^ The X-ray absorption spectroscopy (XAS) spectrum consists of the X-ray absorption near-edge structure (XANES) at low energy and the extended X-ray absorption fine structure (EXAFS) at high energy. While quantitative analysis of the EXAFS is relatively mature, analysis of the XANES is challenging due to its sensitivity to many factors including coordination number (CN),^4^^,^^5^ orbital hybridization,^6^ spin state,^7^ oxidation state,^8^ and symmetry^9^ of the central absorbing atoms. However, the XANES signal usually dominates the XAS and, in principle, provides richer information regarding the coordination environments compared with EXAFS.

A typical analysis of XANES relies on comparisons between experimentally measured spectra from well-known compounds.^10^^,^^11^ There have been attempts at quantitative interpretations of XANES spectra using principal component analysis^12^, ^13^, ^14^ and linear deconvolution methods.^15^ These approaches seek to break down the XANES of a multi-component system into individual component spectra, which provide the statistical basis for estimating the presence and ratios of individual species. However, these techniques are difficult to apply to systems that do not have well-established reference spectra. Theoretical calculations based on time-dependent density functional theory (DFT),^16^ full multiple scattering (FMS),^17^^,^^18^ and Bethe-Salpeter equation approaches^19^ provide an alternative means of obtaining the XANES of any material. Recently, we have developed the first-of-its-kind large, public database of X-ray absorption spectra (XASDB).^20^^,^^21^ Based on the FEFF FMS code,^18^ 580,000 K-edge XANES spectra of over 52,000 crystals in the Materials Project have been calculated and are freely available in the XASDB at the time of writing.^22^ This database not only provides an important reference for experiments but also opens new avenues for large-scale quantitative XANES analysis. For example, we have previously shown that an ensemble-learning spectra matching algorithm can achieve an 84.2% accuracy in identifying oxidation state and local environment by matching unknown spectra with computed spectra in the XASDB.^20^

The extraction of coordination environment information from the XANES is akin to that of image recognition, a field in which machine-learning (ML) techniques have made great strides. Indeed, there have been attempts to apply ML to quantitative and qualitative XANES analysis. For example, Timoshenko et al.^23^ have demonstrated that neural networks can predict the CN of Pt atoms from L-edge XANES spectra of metallic nanoparticles. Carbone et al.^24^ have also shown that convolutional neural networks (CNNs) can predict the coordination environments of 3*d* transition-metal species from site-specific K-edge XANES with an impressive accuracy of 86%. However, the work focused on three types of well-defined coordination, i.e., tetrahedral, square pyramidal, and octahedral, and as acknowledged by the authors themselves, the dominant octahedral environment makes up 64% of the total data. In addition, previous works have reported that material information, such as chemical, elemental, and geometric information, can be obtained from the interpretation of calculated oxygen K-edges ELNES/XANES spectra of metal oxides and SiO_2_ using decision-tree methods.^25^ Very recently, Suzuki et al.^26^ have used L-edge XANES or electron energy loss spectra of MnO in conjunction with a regression model to capture crystal-field parameters.

Despite these advances, two crucial gaps remain. The main limitation is that previous works treated coordination environment identification as a classification problem between mutually exclusive labels. In reality, the coordination environment can be represented along a continuum. For instance, when a species in a perfect regular octahedron is displaced toward one of the vertices, its coordination environment becomes increasingly square-pyramidal-like but still retains features of octahedral coordination. A rigorous treatment of coordination environment therefore needs to define how “square-pyramidal-like” and “octahedron-like” the coordination environment is. A second major limitation is that previous works focus either on a very narrow set of chemistries or environments using experimental XANES data^23^ or a somewhat broader set of chemistries and environments using computed XANES data only.^24^ Given the well-known errors in computed lattice parameters and XANES, it is unclear how ML models trained on large and diverse computed XANES can be applied to experimental XANES.

**Figure 1:**
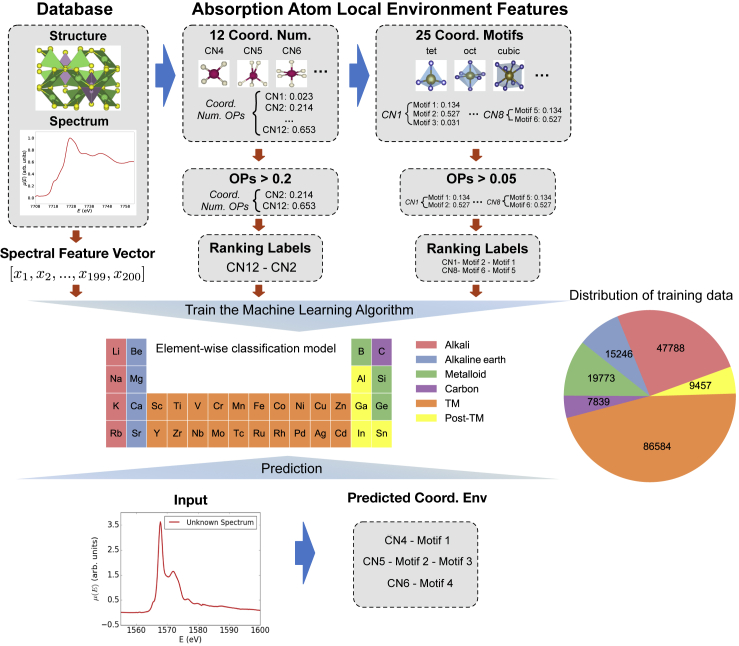
Workflow of the Coordination Environment Identification Algorithm

In this work, we comprehensively address the aforementioned limitations and develop an approach to identify local environments in oxides from K-edge XANES using random forest models. A random forest classification model is an ensemble model in which a multitude of decision trees are constructed by using different subsets of the original data, and the model averages the output from the individual trees to improve model accuracy and reduce overfitting. In contrast to prior models, CNs up to 12, and a total of 25 distinct coordination motifs (CMs), which are enumerated in Figure S1, are considered. It should be noted that while the 25 CMs provide a reasonably thorough description of local geometry in crystals, they are not exhaustive. The model accuracy is assessed by correctly predicting the ranking of the coordination environments with their probabilities above a certain threshold, for example, predicting a six-coordinated atom to have octahedral, pentagonal pyramidal, and hexagonal planar, in decreasing probability. This is a much more comprehensive yet difficult problem to solve than predicting a single CM; correctly predicting only the dominant CM (e.g., octahedral), but not the secondary CMs will still be classified as an inaccurate prediction under our definition. High prediction accuracy of ~85.4% was achieved over 33 cations in oxides, covering most technologically relevant cation species including alkali, alkaline, metalloid, transition metals, post-transition metals, and carbon (Figure 1). Most importantly, we demonstrate the augmentation of the training data with broadened/compressed spectra to mimic the effect of DFT lattice parameter prediction error on spectra. The resulting models can be directly applied to identify coordination environments from experimental XANES with minimal loss of accuracy.

## Results

### Dataset Construction

The training data were constructed from the XASDB of ~190,000 site-specific K-edge XANES of ~22,500 oxides in the Materials Project.^20^, ^21^, ^22^ To the authors' best knowledge, our dataset represents the broadest coverage of cation elements to date in the study of XANES. Figure 1 provides a summary of the total dataset used in this work. Cation elements with atomic number greater than 52 were excluded due to the lack of distinguishable K-edge spectral features. From each spectrum, an energy window of −5 eV to 45 eV from the spectral absorption edge was extracted and converted to a vector of 200 intensity values using linear interpolation. The edge energy (*E*_0_) was determined from the maximum of the first-order derivative. This is the strong scattering XANES region covering the pre-edge, main-peak, and post-peak spectral features.^27^ All three regions have been shown to be critical for the identification of local coordination environments.^24^ The intensity vector was then normalized such that the maximum intensity has a value of 1. The experimentally measurable structure-wise spectra, i.e., the average of all spectra for a particular absorbing element in a structure, were also included in the training data.

In our previous work,^20^ we found that the broadness of the computed XANES feature is sensitive to the lattice parameter. The lattice parameters and bond-length effects on the XANES features have been previously investigated by Timoshenko et al.^28^^,^^29^ whereby bond lengths were varied and subsequent calculations were performed on the distorted structures. In our case, we have found that simple spectra augmentation by compressing or broadening the spectra achieves similar results.^20^ To improve the robustness of the classification models, we split the initial dataset into 80% training and 20% test data and further augmented the training dataset by randomly sampling 30% of spectra and applying broadening or compression of ±5 eV in energy range to mimic the variations in feature broadness. This spectral-shape distortion corresponds to up to 7% variation in the lattice parameters, which exceeds the ~5% systematic errors introduced by the Perdew-Berke-Ernzerhof (PBE)^30^ generalized gradient approximation function used in the Materials Projects for crystal structure optimization.

For each site, the coordination environment is defined as the combination of the CN and the CM. Figure S1 provides a comprehensive enumeration of the CMs considered in this work. The number of spectra for each element is shown in Figure S2. Coordination environment determination for a known structure was carried out using the algorithm by Zimmermann et al.,^31^ as implemented in pymatgen^32^ and matminer.^33^ The algorithm consists of two steps. The first step identifies the number of bonded neighbors to an atom based on the Voronoi tessellation method. The solid angle weights of all neighbors are used to determine a site CN order parameter (OP) that describes how consistent a site is with a certain CN. The CN OP values range from 0 to 1, with 1 representing perfect resemblance. An OP vector $\vec{p}$ is constructed for each site for CNs ranging from 1 to 12, as follows:

(Equation 1) $$\vec{p}=\left\{p_{1},p_{2},p_{3},p_{4},\ldots ,p_{12}\right\},\;\;\text{where}\sum_{i=1}^{12}p_{i}=1\text{,}$$

where *p*_*i*_ denotes the OP for a CN of *i*. CNs greater than 12 are not considered due to their extremely low counts in the dataset, as shown in Figure S3. $\vec{p}$ is a more robust statistical representation of a CN compared with using a single CN value. For example, a site may have *p*_4_ = 0.2 and *p*_6_ = 0.8, indicating that it mostly resembles a CN of 6 and shares some similarity with a CN of 4. This is in contrast to a single-valued CN that is sensitive to radius cutoffs used to determine neighbors and classification. In practice, the CN labels are generated by setting a cutoff for *p*_*i*_ and then concatenating the probability-sorted CNs (see Figure 1). In the second step, the CM is determined by matching the neighbors identified in the first step to prototype motifs. For example, the 6-fold coordination can result from hexagonal planar, octahedral, and pentagonal pyramidal coordination. Again, a vector of OPs $\vec{q}$ based on 25 prototype motifs is computed for each site, as follows:

$$\vec{q}=\left\{q_{\text{single bond}}\times p_{1},\ldots ,q_{\text{tetrahedron}}\times p_{4},q_{\text{octahedron}}\times p_{6},q_{\text{hexagonal planar}}\times p_{6},q_{\text{pentagonal pyramidal}}\times p_{6},\ldots ,q_{\text{cuboctahedra}}\times p_{12}\right\}\text{,}$$

where *q*_*i*_ denotes the OP for a CM prototype of *i*. The CN OPs are factored into the vector of CM OPs $\vec{q}$. The CMs are not mutually exclusive and hence their OP sum will not be 1. In this step, we did not consider CN9, CN10, and CN11, since they do not have dedicated CMs. Similarly, the CM labels are generated by setting a threshold for CM and concatenating the probability-sorted CMs, as shown in Figure 1. Our strategy of using ranking labels provides a rich representation of the coordination environment. The ranking labels of CM OPs were encoded for a specific type of CN. For example, we took into account only $\left\{q_{\text{octahedron}}\times p_{6},q_{\text{hexagonal planar}}\times p_{6},q_{\text{pentagonal pyramidal}}\times p_{6}\right\}$ for generating CM ranking label of CN = 6 (see Experimental Procedures for details).

The coordination environment classification task can then be divided into two sequential steps powered by two separate models for each element. In the first step, the CN model identifies the CN ranking label from the spectra, and in the second step the CM model identifies the CN-specific CM ranking label. The models are trained for each element as the characteristic XAS absorption edge energy follows a power law with atomic number and is well separated.^34^ The absorbing species can be identified with 100% accuracy from simply examining the spectral energy range. This domain knowledge significantly reduces the problem complexity and is expected to improve model accuracy. Eventually, the coordination environment recognition problem becomes a two-step multi-label classification problem, whereby an absorption spectrum might reflect a statistical ensemble of more than one coordination environment. This is an attractive problem transformation approach that provides both scalability and flexibility^35^ to handle most off-the-shelf multi-label classification algorithms.^36^, ^37^, ^38^

### Machine-Learning Models

Figure 1 provides an overview of the coordination environment classification workflow. As some elements are found only in specific local environments,^39^ the knowledge of elemental types would already significantly narrow the range of possible local environments. Indeed, a “baseline” model can be constructed that merely assigns a CN-CM classification based on the dominant environment for that element. Such a baseline model has a high classification accuracy of 70%–80% on the first-row transition-metal cations from Sc to Ni, an intermediate accuracy of ~60% for the post-transition metals and metalloid, and a relatively low accuracy of 17%–58% for the alkali and alkaline earth cations (see Figure 3). Any reasonable ML models, therefore, have to achieve substantial improvements over this “baseline” model across all chemical classes.

In the next steps, optimized element-specific ML models sequentially identify firstly the CN ranking label, followed by the CN-specific CM ranking label, from the spectra. Five ML models were assessed in terms of the performance in CN and CM classification, namely *k*-nearest neighbor (*k*NN), random forest, multi-layer perceptron (MLP),^40^ CNN,^40^ and support vector classifier (SVC). Five-fold cross-validation was used for model fitting and hyper-parameter optimization. During the optimization process, we performed a grid search to identify optimal values for key ML parameters that are directly related to the classifiers' performances. These parameters include *k* in the *k*NN model, number of trees in the random forest model, number of neuron/layers and choice of activation function in MLP and CNN, and the penalty parameter *C* and the kernel coefficient (*γ*) for the SVC. For all the other parameters, we used the defaults within the scikit-learn package.^38^ Previous works have shown that the performance of the CNN-based model in the classification of XAS is insensitive across different neural network structures.^24^ The same hyper-parameter space was adopted in the optimization of ML models for each classification subtask (see Experimental Procedures for details).

As shown in Figure 1, this work focuses only on elements in rows 2–5 of the periodic table, excluding the noble gases; elements in row 6 and beyond, including the rare earth elements, were not investigated because of the lack of resolution in the K-edge absorption spectra for elements with atomic number greater than 52.

### Computational Spectra Classification Performance

**Figure 2:**
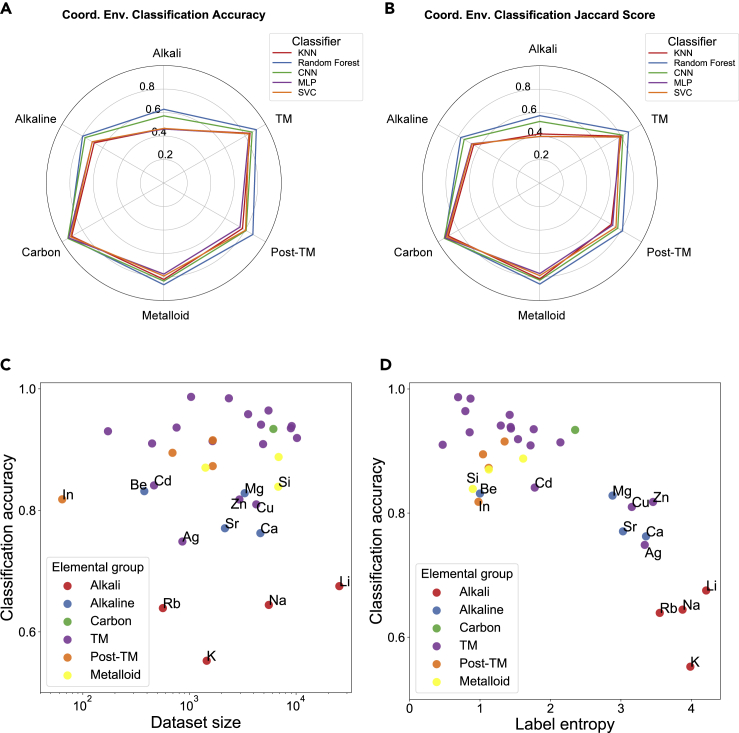
Performance of Five ML Classifiers—*k*NN, Random Forest, CNN, MLP, and SVC—on Coordination Environment Classification (A and B) Accuracy (A) and Jaccard score (B) for the five ML classifiers broken down by elemental categories, namely alkali metals, alkaline earth metals, transition metals (TM), post-transition metals, metalloids, and carbon (see Figure 1 for color-coded categories). (C) Relationship between the random forest model's classification accuracy and the dataset size. (D) Relationship between the random forest model's classification accuracy and the training label entropy. *k*NN, *k-*nearest neighbor; CNN, convolutional neural networks; MLP, multi-layer perceptron; SVC, support vector classifier. Cation elements with classification accuracy less than 0.85 are labeled in (C) and (D).

Figures 2A and 2B compare the accuracy and Jaccard index (see Experimental Procedures for definitions), respectively, of the optimized five classifiers broken down into the six elemental categories. The accuracy captures how well each ML model performs in predicting the top-ranked coordination environment, i.e., the combined CN-CM score with the highest value. The Jaccard index, on the other hand, captures how well each ML model performs in identifying all relevant coordination environments related to the absorbing species, i.e., all CN and CM with non-zero OPs. See Experimental Procedures for all element categories the random forest classifiers outperform the other classifiers, with an overall accuracy of 85.4% and a Jaccard score of 81.8%.

One key observation from Figures 2A and 2B is that classification performance is highly dependent on the elemental category. While the performances of all classifiers are relatively high (>90% accuracy) for carbon, the performances on the alkali metals are comparatively poor. To elucidate the origin of the performance variations, we have plotted the classification accuracy for the best-performing random forest model against training dataset size and label entropy in Figures 2C and 2D, respectively. Here, the label entropy,^41^ which is an informational measure of the diversity of the coordination environment labels in each elemental category, is computed using the following expression:

(Equation 2) $$S=-\sum_{i}P_{i}{\text{log}}_{2}P_{i}\text{,}$$

where *P*_*i*_ is the probability of a ranking label *i* out of all ranking labels. The label entropy *S* is high if the variability of the label values is high, i.e., an element exists in a spectrum of coordination environments with similar probabilities. For example, the alkali metals Li, Na, and K have high label entropy because they exist in a variety of local environments—tetrahedral, octahedral—with relatively high probabilities, while the transition metals have low label entropy because they exist mainly in the octahedral coordination, with the exception of the higher oxidation states of V and Cr that almost always exist in tetrahedral coordination.^39^ The Jaccard index with data size and label entropy is shown in Figure S4, which shows a trend similar to that of the accuracy.

From Figure 2C, it may be observed that there is no clear relationship between classifier performance and training dataset size. However, a clear inverse relationship between classifier performance and the label entropy can be seen in Figure 2D. These observations suggest that data size is not the dominating factor, and the current data size for each element seems sufficient to reach convergent results. The decrease in performance with an increase in label entropy is expected, given that it is much more challenging for a classifier to distinguish between several equi-probable environments as opposed to identifying a single dominant label. This explains the especially poor performance on the light alkali elements (Li, Na, and K). In this case, the increase in training dataset size generally leads to an increase in the classification accuracy. For example, the label entropy values of all three light alkali cation elements are all close to 4, while their dataset sizes differ greatly. The training dataset size (25,450) of Li is one magnitude higher than the training dataset size (1,451) of K, and the classification accuracy of Li is 0.12 higher than K. For alkaline earth metals (Be, Mg, Ca, Sr) the coordination environment becomes more diverse as the ionic radius increases, and performance drops accordingly. In the dataset, Be^2+^ is always four-coordinated while Mg^2+^, Ca^2+^, and Sr^2+^ are found to be four-, five-, six-, seven-, or eight-coordinated.

As a comparison, Figure S5 shows CNN's prediction accuracy as a function of label entropy values. The CNN classifier fails to deliver classification performances comparable with the random forest classifier. This can be attributed to the relatively small data size per element-CM, with an average of ~110 (Figure S6), since it is known that neural networks-based models generally need more data to train. Unsurprisingly, CNN model performance shows a more notable positive relationship with the data size (Figure S5B). In addition, the CNN classifier shows a greater decrease in prediction accuracy as label entropy increases.

**Figure 3:**
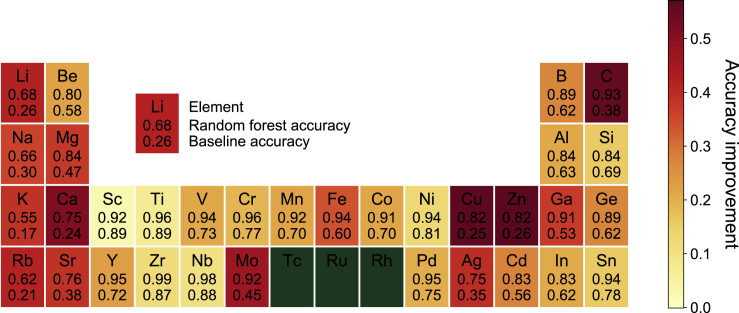
Comparison of Accuracy of Optimized Random Forest Models with the Baseline Model for All Elements Studied In general, the random forest models outperform the baseline model by significant margins (color of rectangles indicates the level of improvement). Tc, Ru, and Rh are excluded due to the lack of data.

Figure 3 shows a comparison of the accuracy of the random forest models with the “baseline” models. The accuracy of the random forest models are well over 80% for the majority of elements and exceeds 55% even in the more challenging alkali elements. In general, the random forest models far outperform the “baseline” models. High Jaccard indexes are also achieved across the periodic table, as shown in Figure S7.

### Coordination Environment Identification from Experimental XANES Spectra

We evaluated the random forest classifiers using 28 high-quality normalized XANES experimental spectra obtained from the XAFS Spectra Library^42^ and EELS database,^43^ supplemented by six high-quality experimental XANES spectra of V_2_O_5_, V_2_O_3_, VO_2_, LiNiO_2_, LiCoO_2_, and NiO from previous studies.^44^^,^^45^ These 28 spectra comprise a diverse dataset covering 13 chemical species for classifiers' performance assessment. For spectra from the EELS database and XAFS Spectra Library without available structural information, we assumed that they correspond to the ground-state structures in the Materials Project database with the same chemical composition.

We selected the spectral region from −5 eV to 45 eV with reference to edge energy (*E*_0_) determined by the MBACK algorithm.^46^ As the PBE functional usually leads to up to 5% lattice parameter overestimation error,^47^^,^^48^ the expanded spectral region encompasses this artificial spectral feature difference between computational and experimental XANES. We used linear interpolation to convert the experimental spectra to vectors of 200 intensity values and normalized them to the maximum intensity value. It should be stressed, however, that the experimental spectra were not used in the training of the random forest models.

The random forest classifier successfully identified 23 of the 28 top coordination environment ranking labels, with a coordination environment prediction accuracy of 82.1% and a Jaccard score of 80.4%. These accuracies are comparable with those achieved on the computational test set. The random forest classifiers failed to predict the correct coordination environment for two phases of V_2_O_5_, ZnO, Na_2_O, and CuO from the experimental spectra, although the models predicted the dominant CN (CN with highest *p*_CN_) with 100% accuracy. For V_2_O_5_, the classifier successfully predicts the dominant CM, i.e., trigonal bipyramidal, but does not predict the correct order of secondary and tertiary CMs (a failure by our strict definition). The likely reason for this failure is the small difference in OPs between the second (i.e., *q*_pentagonal planar_) and third (i.e., *q*_square, pyramidal_) ranked CMs of ~0.029. In ZnO, the coordination environment of Zn does not resemble any target CMs, i.e., all CM OPs are <0.22. Here, the relatively low resemblance between the absorbing atom's coordination pattern and target motifs seems to be the critical issue. For Na_2_O, the failure of the model may be attributed to the possible contamination of the experimental sample.^20^ Finally, for CuO, the Cu^2+^ has a four-fold coordination with oxygen that is matched with five target motifs. The OPs of three of the matched CMs—rectangular see-saw-like, see-saw-like, and square co−planar—exceed 0.5. In this case, the use of EXAFS may be required to identify the local environment with sufficient resolution.

### Model Insights

We performed feature importance analysis to gain insights into the contribution of different regions of the K-edge XANES spectra to coordination environment information. The studied cases include CN = 2–8 for all 33 elements in this work. We divided each K-edge XANES spectrum into three regions, the pre-edge, main-peak, and post-peak, with energy ranges of 0–15 eV, 15–30 eV and 30–45 eV, respectively, referenced to the spectral onset. A robust brute-force drop-variable importance approach was used, whereby part of the input features was systematically dropped to assess the change in model prediction accuracy. In this approach, a baseline model was first trained using the entire spectra. Each spectrum was then divided into several regions and certain regions were dropped from the spectrum. The remaining incomplete spectra were used to train new models. In principle, dropping more important regions would lead to poorer model performance. The advantage of the drop-variable importance measure is that it provides the ground truth feature importance compared with alternative importance measures.^49^ Both single and combined regions, i.e., “Pre + Main”, “Pre + Post.” and “Main + Post,” were investigated.

**Figure 4:**
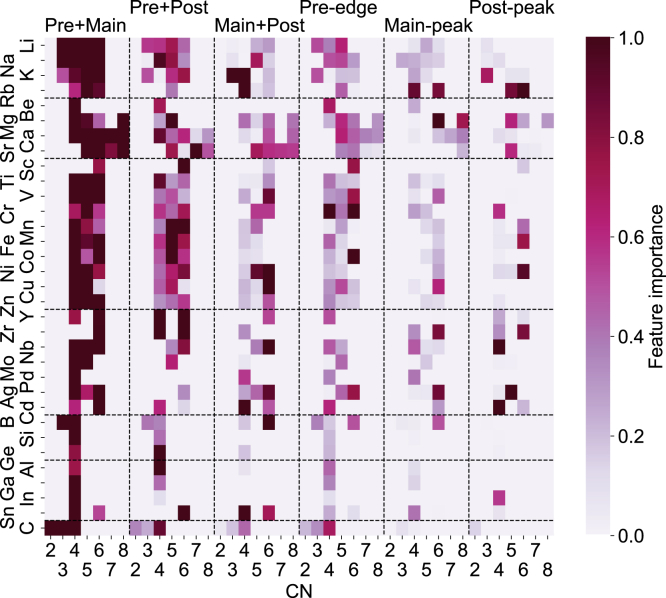
Normalized Feature Importance of Different Regions of Spectra for Predicting a Given CN for Each Element The drop-variable feature importance is normalized with respect to the maximum importance of a spectral region for each element. The x axis is arranged by spectral regions (i.e., pre + main, pre + post, and so forth) followed by increasing CN within each spectral region. The y axis is arranged by elemental category (i.e., starting from the top, alkali, alkaline earth, 3d TM, 4d TM, metalloid, post-TM, and C) followed by ascending atomic numbers in each elemental category.

The normalized spectral regional feature importance of all elements in predicting certain CN is shown in Figure 4. The x axis denotes the CN grouped by the spectral region as shown by the labels on the top of the graph, and the y axis shows the grouped elements. For elements that do not have certain CNs, the feature importance is set to 0. Unsurprisingly, the “Pre + Main” region of the features plays a key role in all corresponding CNs and, in general, joint spectral regions have higher feature importance than single ones. The high feature importance for joint spectral regions implies that full spectral characteristics are necessary for accurate coordination environment identification, consistently with previous studies.^24^ Even for CN4, the highest feature importance is achieved using “Pre + Main” spectral regions followed by “Pre + Post.” In addition, “Main + Post” becomes more important with increasing CN, in good agreement with previous studies.^8^^,^^4^^,^^24^

**Figure 5:**
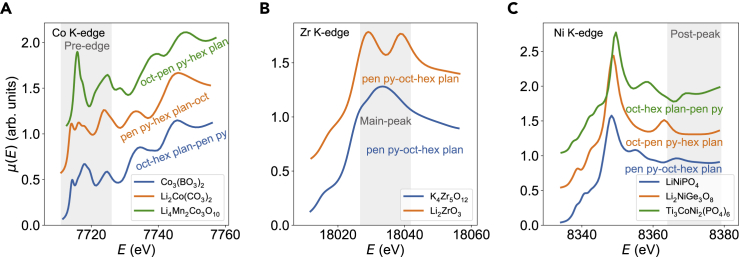
Feature Importance Examples for Six-Coordinated Transition Metals The most important spectral regions for Co (A), Zr (B), and Ni (C) are pre-edge, main-peak, and post-peak, respectively. The top CM label is annotated.

For the first-row (3*d*) transition metals, the pre-edge plays an important role. This is due to the well-known fact that 3*d* transition metals with tetrahedral geometries tend to have strong pre-edge intensity due to the hybridization of unoccupied *p* and *d* states.^50^^,^^51^ In addition, the early 3*d* transition metals tend to have stronger pre-edge effects than late ones. Our data-driven approach is able to capture this relationship known from group theory analysis. Figure 5 provides an illustration of how the feature importance can be observed in the K-edge XANES for various six-coordinated transition metals. In Co, Zr, and Ni, changes in the local environment predominantly affect the pre-edge, main-peak, and post-peak regions, respectively.

## Discussion

In summary, we have demonstrated that random forest models trained on FEFF-computed K-edge XANES can be used to directly predict the coordination environment—both CN and CM—with high accuracy. In contrast to prior works, we eschew a rigid classification of coordination environments into mutually exclusive labels, opting instead for a more rigorous, mathematical definition of coordination environment based on multiple labels with order parameters.

Prior works on identifying coordination environments from XANES have primarily focused on deep-learning models, i.e., MLP and CNN.^23^^,^^24^ While such deep-learning models perform respectably, especially for transition metals, one major finding of our work is that the random forest models outperform them by significant margins. The likely reason is that deep-learning models are notoriously data hungry; for many elements, there is insufficient data to train such models properly. On the other hand, the accuracy of the optimized random forest models show little to weak dependence on data size and much stronger dependence on label entropy, suggesting that the random forest models are limited by task difficulty as opposed to data limitations.

We also strongly advocate the development of baseline models for the evaluation of ML models in materials science. The commonly used “accuracy” metric means little without this context. As is evident from Figure 3, many elements, the 3*d* transition metals being a notable category, have very high probabilities of being in a particular coordination environment, i.e., information content/label entropy is low. For instance, any ML model that achieves anything less than 89% accuracy in classifying the coordination environment of Ti is, in effect, underperforming relative to a trivial model that always identifies Ti as being in a six-coordinated octahedron environment. Indeed, the random forest models yield far more substantial accuracy improvements in coordination environment identification over the baseline model for elements that exist in many different environments with high probability, e.g., Cu, Zn, C, and the alkali metals. For the 3*d* transition metals, the accuracy improvement is a relatively modest ~20%, even though high absolute accuracies exceeding 90% are achieved in all instances.

Most importantly, we demonstrate a data-augmentation approach that enables the random forest models trained on computed data to be directly applied to experimental K-edge XANES with minimal loss in accuracy. The models achieved an outstanding accuracy of ~82.1% in identifying the dominant coordination environment over a diverse experimental spectra test set comprising 28 experimental K-edge XANES of 13 chemical species. To illustrate the importance of augmenting the site-specific training data with site-averaged spectra and broadened/compressed spectra, we have constructed random forest models with and without the inclusion of site-averaged spectra and with and without the inclusion of broadened/compressed data. Table 1 shows the performance of the different models. Among the four types of spectra sources, the highest performance is achieved when both site-specific and site-averaged spectra augmented with distorted spectra are used. Interestingly, the model performance suffers with data augmentation when only site-specific data are used. We surmise that this is due to the fact that site-specific spectra are very different from site-averaged spectra (what experiments measure) for most structures, which have more than one symmetrically distinct site for an element. We have also performed tests of the random forest models' performance on the energy resolution and presence of noise in the experimental spectra. As can be seen from Figure S8, the performance is generally robust against changes in energy resolution and noise.

**Table 1:** Classification Accuracy and Jaccard Score of Experimental Dataset with Different Training Spectra Source and whether the Training Data Are Augmented with Broadened/Compressed Spectra

Spectra Source	Augmented?	Accuracy	Jaccard Score
Site and averaged	yes	0.821	0.804
Site and averaged	no	0.786	0.768
Site-specific	yes	0.643	0.625
Site-specific	no	0.714	0.696

The site-specific spectra are direct outputs from the calculations and the averaged spectra are site-averaged structure-wise spectra.

This work addresses a critical gap in ML-based K-edge XANES analysis. High-quality experimental XANES data are difficult and expensive to obtain (see the excellent review by Asakura et al.^52^ on the challenges in constructing an international XAFS database). High-throughput computations are currently the only approach to generate large and diverse XANES datasets. Being able to develop a coordination environment identification ML model using the latter that can be applied to the former is therefore of major value, and represents a transformative advance in the application of ML to coordination environment identification. We further note that the existing ML models represent a proof of concept and can always be retrained with the inclusion of experimental XANES from high-throughput XAS experiments when they become available. We expect that these future developments will further increase the accuracy of the models.

We conclude by noting a few limitations in the current work. First, XANES is commonly used to characterize amorphous materials and disordered crystals, as well as ordered crystals. In amorphous materials, the atomic coordination environments are highly diverse, i.e., very high label entropy. We therefore do not expect the current ML models trained on crystalline local environments to perform well on such materials. We do, however, expect the ML models to perform reasonably well on disordered crystals, insofar as the local environments in the disordered crystals are represented within the existing data, which comprise mainly relaxed ordered crystals from the Materials Project. Second, the set of CMs used in the present work is not exhaustive and limited by the algorithm used—e.g., the CN6 trigonal prismatic motif is not present. Nevertheless, we fully expect that this limitation can be addressed with the inclusion of order parameters for additional motifs in future and retraining of the models. Finally, although temperature can have a pronounced effect on XANES,^53^ we did not explicitly consider its effect in this work, although we expect that the data-augmentation procedure would have accounted for it to some extent.

## Experimental Procedures

### Construction of Coordination Environment Ranking Labels

Given a real-valued vector $\hat{\mathrm{OP}}\in R^{L}$, the *i*th OP represents how closely the site's local coordination environment resembles a CN condition or a specific CM. A threshold *t* is applied to $\hat{OP}s$ to create a bipartition of relevant and irrelevant CN and CM labels. The multi-label prediction $\hat{y}$ can be obtained as

(Equation 3) $${\hat{y}}_{j}=\left\{ \begin{array}{cc} 1 & \text{if}\;\;{\hat{OP}}_{j}\geq t\\ 0 & \text{if }\;{\hat{OP}}_{j}<t \end{array}\right.\text{.}$$

Instead of using an arbitrary threshold like 0.5, we adopted the concept of label cardinality (LCard) and calibrated the threshold *t* to minimize the possibility of a spectrum being assigned to the no-label set. The LCard^54^ is a standard measure of “multi-labeled-ness,” which is simply the average number of labels associated with each example. For *N* examples and *L* labels, the LCard measure can be calculated as:

(Equation 4) $$\text{LCard}=\frac{1}{N}\sum_{i=1}^{N}\sum_{j=1}^{L}y_{j}^{i}\text{.}$$

The threshold *t*_1_ for CN and threshold *t*_2_ for CM were calibrated using the same procedure, as follows:

(Equation 5) $$t=\underset{t}{\mathrm{argmin}}\left\|\text{LCard}\left(D_{\text{site}-\text{specific}}\right)-\text{LCard}\left(D_{\text{site}-\text{averaged}}\right)\right\|\text{,}$$

where *D*_site-specific_ and *D*_site-averaged_ are the dataset of ~110,000 site-specific and ~36,000 site-averaged computed K-edge XANES spectra, respectively. The site-averaged spectral dataset was also considered here, as experimentally measured XANES spectra are the averaged absorption coefficients. The OPs of site-averaged spectra were obtained by averaging site-specific OPs of the same element. The calibration procedure aims at minimizing the difference between label cardinality of site-specific spectra and that of site-averaged spectra. This calibration approach has been found to be more effective and efficient in reducing the probability of empty-set prediction issues.^35^

We evaluated the threshold value *t*_1_ and *t*_2_ from 0 to 0.4 at 0.01 intervals. The average number of CN labels associated with each spectrum dropped below 1 when *t*_1_ exceeded 0.4, and this was set at the upper limit. For the CN label set, we found that the LCard difference between the site-specific dataset and site-averaged dataset is minimized at *t*_1_ = 0.2. The average number of CN labels associated with each spectral example was ~1.2. For the CM label set, the difference in LCard between the two datasets reaches a minimum at *t*_2_ = 0.05. The average number of coordination environment labels associated with each spectrum was ~3.2.

After applying the calibrated thresholds, we then encoded the CN and CM label sets into the form of ranking labels in terms of descending OPs. Using 0.2 as cutoff for CN OPs, the average number of CN ranking labels per element was 10. Note that the labels contain joint labels such as CN4 to CN6. In the CM classification task, the average number of CM ranking labels is 5 per element per CN. As expected, the distribution of relevant CN labels, i.e., CN with *p*_CN_ ≥ 0.2, was inhomogeneous (Figure S3). For each element, there are a few dominant CNs with an order of magnitude more data points than the other CNs. In the CM classification problem, we therefore restricted our consideration to those most abundant CN cases of each elemental group. Only CNs ≤8 were considered for the CM classification task, as no target CM was provided for CN = 9–11 and only one CM was provided for CN = 12.

For each absorbing species, we excluded CN and CM ranking labels with less than 30 samples. After applying this rule, all Tc, Ru, and Rh ions are six-coordinated. Therefore, we removed the K-edge XANES from the first step CN classification task's training dataset. For the CM classification task, we repeated this operation and excluded those sub-datasets (see Table S1) associated with only one CM label from the training dataset as well. The final CNs in each elemental group that were subjected to the coordination environment classification task are given in Table 2.

**Table 2:** Coordination Number for Each Elemental Group Subjected to Coordination Environment Classification Task

Element Group	CN
Alkali	3–8
Alkaline earth	4–8
Metalloid	3–4
Carbon	2–4
Transition metal	4–6
Post-transition metal	4–6

To validate the necessity of using ranking labels to represent the absorption elements' coordination environments, we visualized the joint distributions of the CN and CM OPs of the alkali and the transition-metal elemental group (Figure S9). From Figure S9, we observe that there are correlations across different CN OPs or CM OPs and that multiple coordination environments coexist. We also note that the correlation between CM OPs is quite substantial and that most six-coordinated transition-metal ions' coordination patterns resemble two or more CMs with OPs exceeding ≥0.4. These findings emphasize that labeling the absorbing sites' coordination environments with one label cannot adequately represent the full coordination environment.

### Hyper-parameter Optimization of Machine-Learning Algorithms

In this work, we use the top-1 accuracy and Jaccard index as metrics to evaluate the performance of classifiers. The top-1 accuracy of a classifier is evaluated by its ability to yield the top-ranked coordination environment.

(Equation 6) $$A=\frac{1}{N}\sum_{n=1}^{N}\left(l_{n}^{1}=={\hat{l}}_{n}^{1}\right)\text{,}$$

where $l_{n}^{1}$ denotes the top-1 label for the *n*th spectrum in a total of *N* spectra, and the ${\hat{l}}_{n}^{1}$ is the estimated top-1 label from models. The same equation is used for element-wise accuracy and the overall accuracy computations.

The Jaccard index measures the overlaps between the true CN-CM labels and the predicted CN-CM labels. Let *y*_*n*_ be the ground true CN-CM label set of the *n*th spectrum and ${\hat{y}}_{n}$ be the predicted CN-CM label made by a classifier. The Jaccard index can be computed based on the number of labels in the intersection set divided by the number of labels in the union set:

(Equation 7) $$J\left(y_{n},{\hat{y}}_{n}\right)=\frac{\left|y_{n}\cap {\hat{y}}_{n}\right|}{\left|y_{n}\cap {\hat{y}}_{n}\right|}\text{.}$$

The Jaccard index yields a number (0%–100%) indicating how well a given classifier identifies all relevant coordination environments compared with the correct coordination environments.

The hyper-parameter spaces investigated for each ML model are as follows:

1 *k*NN: the *k*-nearest neighbors classifier was optimized with respect to the number of neighbors (*N*) and the distance metric (*p*). The values of *N* examined were 10, 20, 30, and 50. The minimum value of *N* = 10 was set to avoid overfitting and increase the generalizability of models. The Manhattan distance and Euclidean distance were used to assess the distance metric effects.
2 Random forest classifier: the number of trees in the forest was tested at values 10, 20, 30, 50, 100, and 200. The rest of the parameters were kept at the default settings of scikit-learn package.^38^
3 MLP: for the MLP classifier, the number of hidden layers (*L*) was varied from 1 to 3 and the number of neurons in each hidden layer was varied from 10 to 100. The activation functions tested were the logistic, tanh, and ReLU functions.
4 SVC: the penalty parameter *C* was drawn exponentially from 0.001 to 100.0. The maximum value of *C* was set at 100.0, as high *C* is prone to overfitting. Two kernel coefficient (*γ*) values were tested: (a) 1 divided by the number of features (*γ* = 0.005) and (b) 1 divided by the number of features multiplied by the variance of the spectral absorption coefficients (*γ* ≃ 0.013). The radial basis function (RBF) kernel was set as the number of observations that is one to two orders of magnitude higher than the number of features in the training data. In addition, a previous study^55^ has shown that it is unnecessary to consider the linear kernel if the model selection is conducted using the RBF kernel.
5 CNN: the two-layer CNN classifier was used. The two layers were fully connected, with feedforward hidden layers with 50 and 100 neurons, ending with a softmax output layer. The number of neurons in the output layer equals the number of target ranking labels.

For CN ranking labels classification, we found that the model using 10 nearest neighbors and Manhattan distance performs the best for *k*NN models. The random forest classifier's performance converged at 30 trees for all elemental groups. For the MLP classifier, the two-layer neural network architecture with ReLU activation function outperformed the rest of the models with tanh or logistic sigmoid neurons. The best MLP model had 50 neurons in the first hidden layer and 100 in the second hidden layer. We found that further increasing number of hidden layers has a detrimental effect on classification performance. For the RBF SVC classifier, the model with *C* = 100 and γ≃0.013 performed the best.

For the CM ranking labels classification task, the optimum CN classifiers' parameter configurations were the best sets for *k*NN classifier, MLP classifier, and RBF SVC classifier as well. We found that the random forest classifier performed the best when the number of trees in the forest equaled 50.

## Data and Code Availability

The K-edge XANES data are available from Materials Project website under the XAS app (https://materialsproject.org/#apps/xas/). The models presented in this work are available in the maml python package (https://github.com/materialsvirtuallab/maml) developed by the Materials Virtual Lab, and the corresponding web app is deployed at https://xas.crystals.ai.

## Supplemental Information

Document S1. Figures S1–S9 and Tables S1 and S2

Document S2. Article plus Supplemental Information
