# Supplementary material for: Random Forest Models for Accurate Identification of Coordination Environments from X-Ray Absorption Near-Edge Structure
Source: Patterns (N Y). 2020 Apr 21;1(2):100013. doi: 10.1016/j.patter.2020.100013 (PMC7660409; doi:10.1016/j.patter.2020.100013)
Supplement: Document S1. Figures S1–S9 and Tables S1 and S2 [file mmc1.pdf]

PATTER, Volume *1*

Supplemental Information

Random Forest Models for Accurate Identification of Coordination Environments from X-Ray Absorption Near-Edge Structure

Chen Zheng, Chi Chen, Yiming Chen, and Shyue Ping Ong

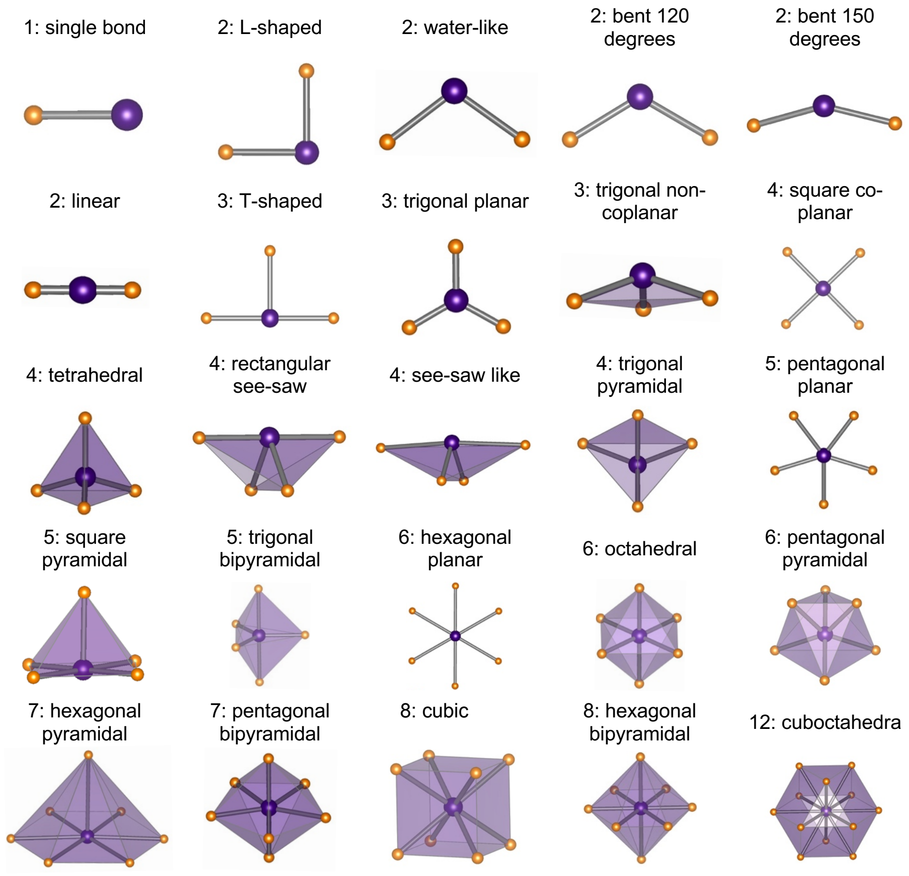

Figure S1: Twenty five coordination environment motifs considered in this work. Each chemical environment is labeled by “coordination number: coordination motif”.

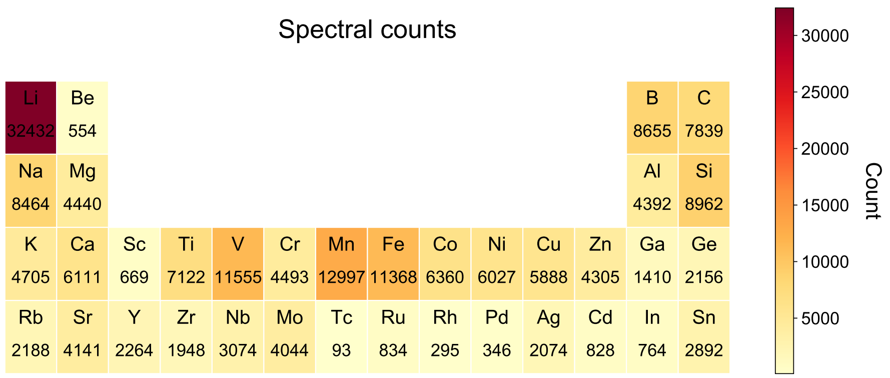

Figure S2: Number of spectra in each element category.

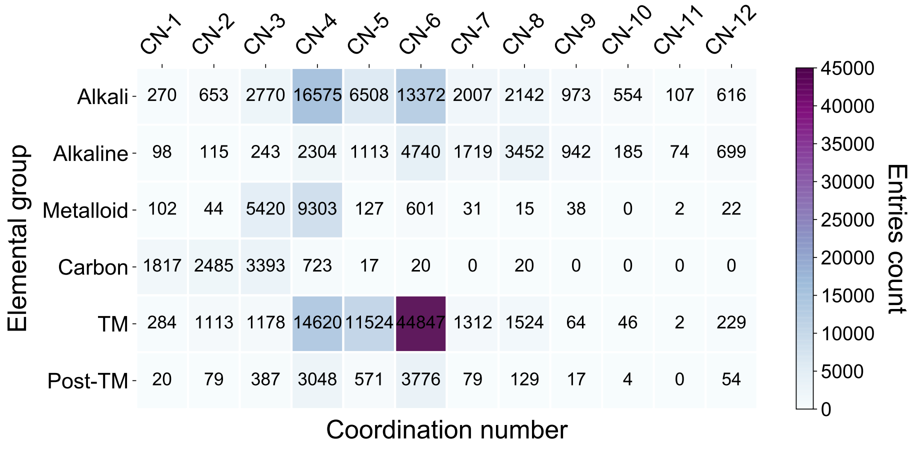

(a) Coordination number from 1 to 12

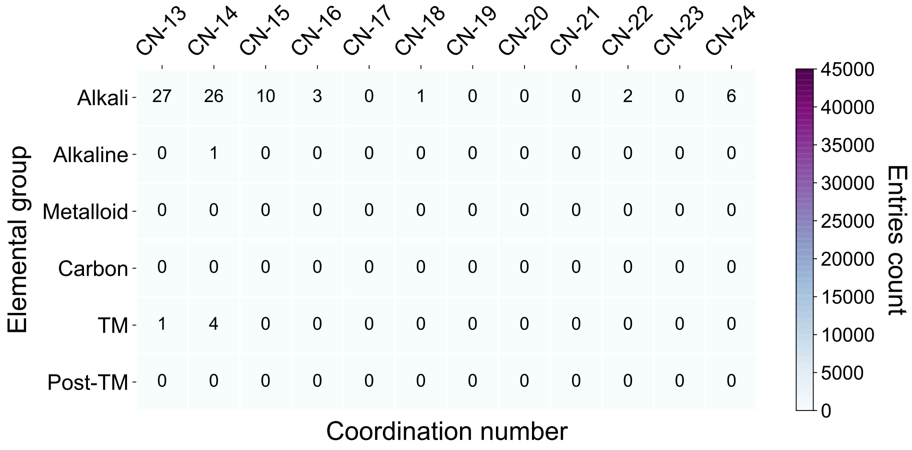

(b) Coordination number from 13 to 24

Figure S3: Number of K-edge XANES entries with coordination number order parameters (OPs) larger than 0.2.

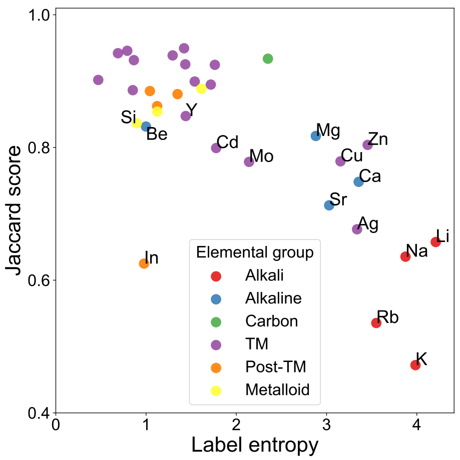

(a)

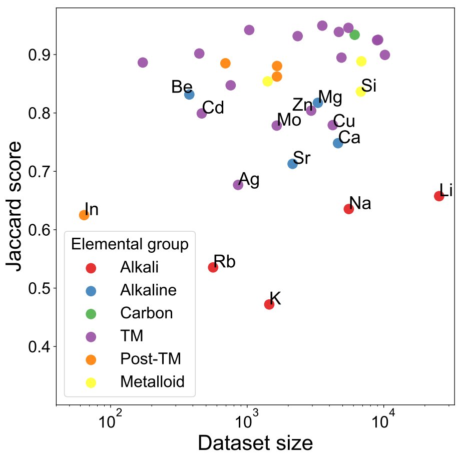

(b)

Figure S4: Jaccard scores of random forest classifier with respect to (a) label entropy and (b) training dataset size categorized by elemental groups.

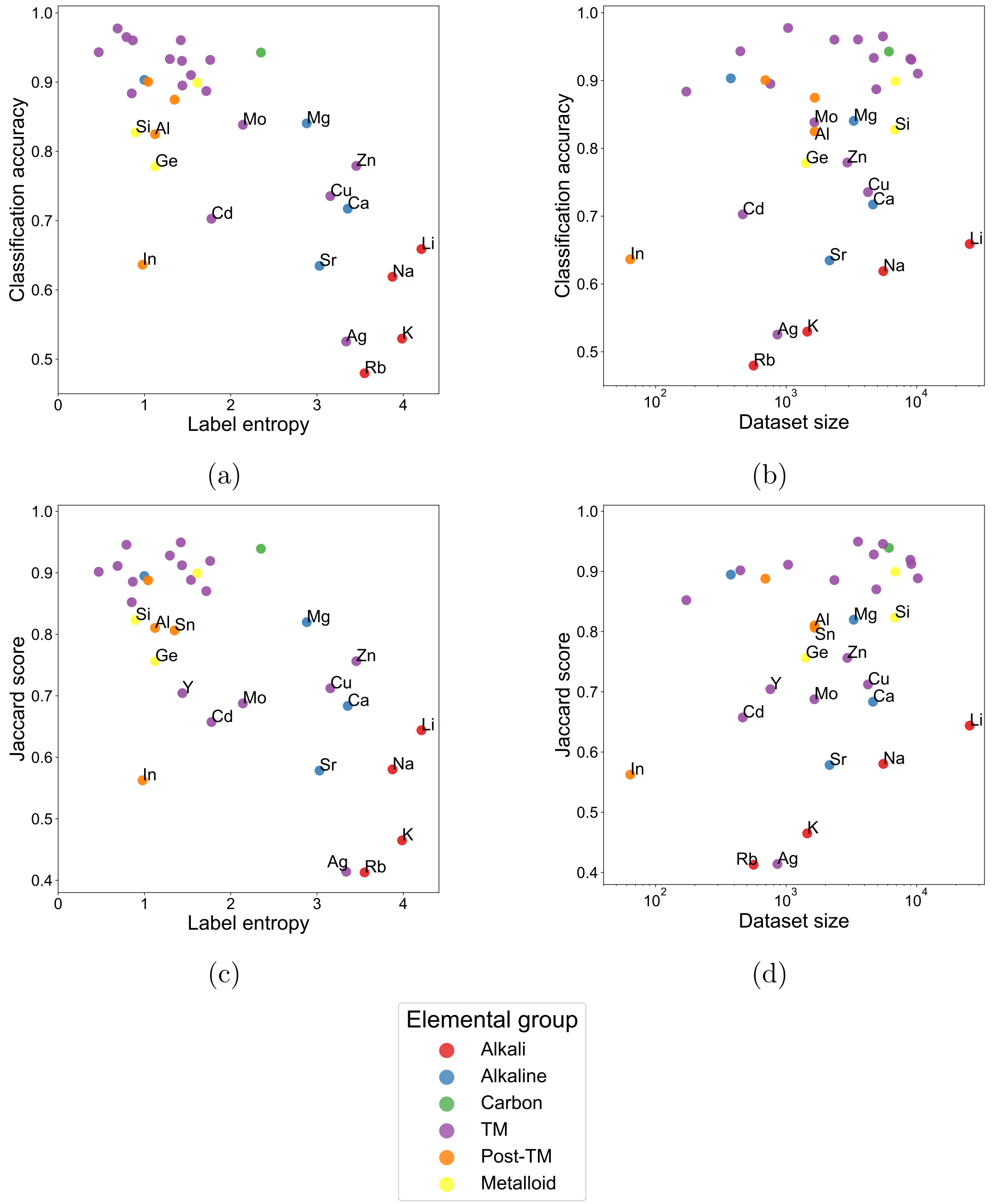

Figure S5: Performance of convolutional neural network classifier with respect to label entropy and training dataset size. Top row plots the relationships between the top coordination environment classification accuracy and (a) label entropy and (b) training dataset size. Bottom row plots the relationships between the Jaccard score and (c) label entropy and (d) training dataset size. Cation elements with classification accuracy less than 0.85 are labelled in the figures.

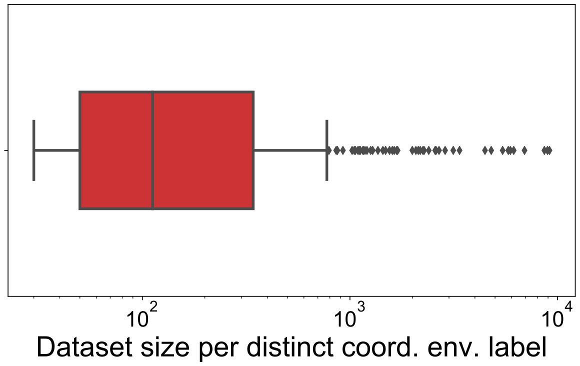

Figure S6: A boxplot of the number of spectra for each element-coordination environment category. The distinct coordination environment label is a combination of element and the coordination environment, e.g., Ti-CN6-octahedron.

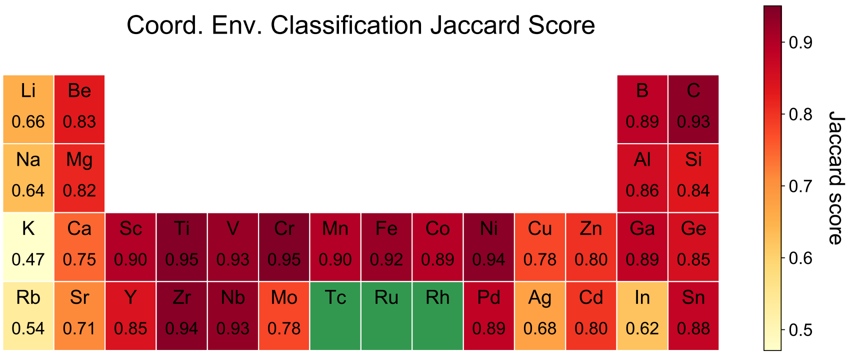

Figure S7: The random forest classifier's element-wise classification Jaccard scores of coordination environment classification. We do not have sufficient computed K-edge XANES for Tc, Ru, and Rh to form a reliable training set for classification tasks.

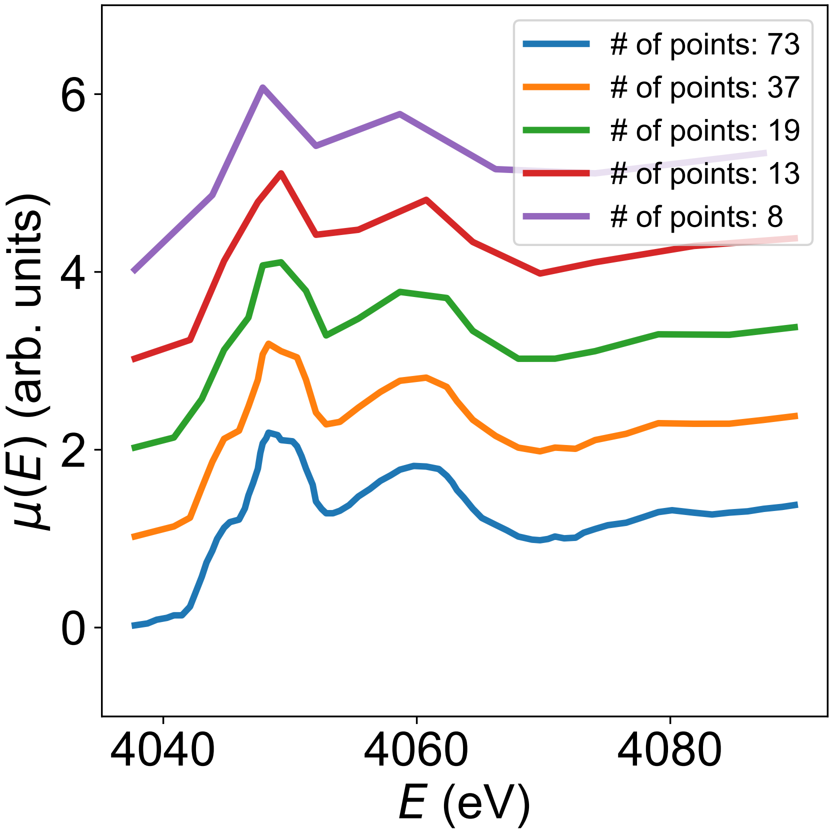

Figure S8: Energy sub-sampled Ca K-edge XANES spectra for $CaCO_3$[1] that has an initial resolution of 73 data points across the energy range 4037.8-4089.8 eV. The sampled spectra have lower resolutions with 8, 13, 19 and 37 data points in the same energy range. We find that the ML model correctly predicts the coordination motif for all resolutions except the lowest one with 8 data points.

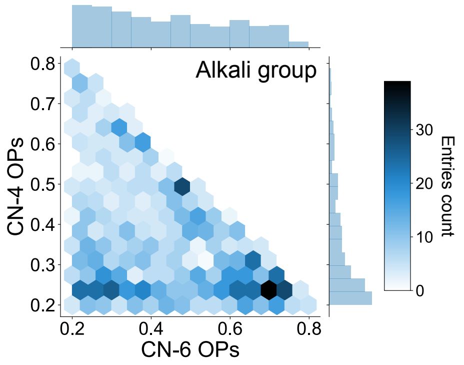

(a) Joint distribution of coordination number OPs of four and six coordinated atoms in alkali metal oxides.

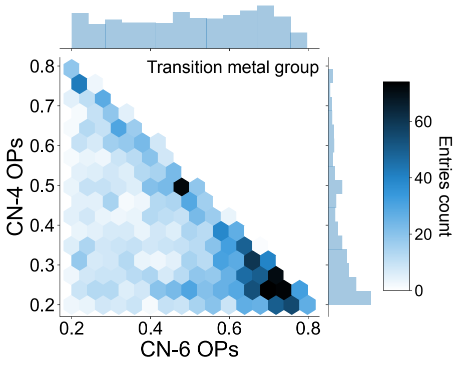

(b) Joint distribution of coordination number OPs of four and six coordinated atoms in transition metal oxides.

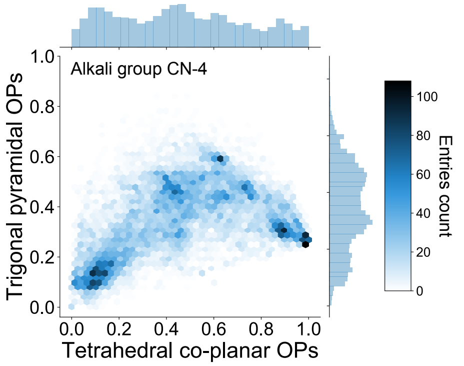

(c) Joint distribution of OPs for trigonal pyramidal and tetrahedral co-planar coordination motifs in four coordinated alkali metal oxides.

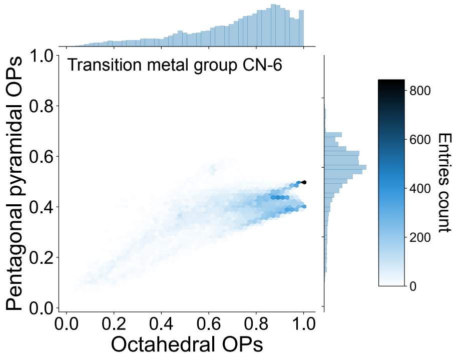

(d) Joint distribution of OPs for pentagonal pyramidal and octahedral coordination motifs in six coordinated transition metal oxides.

Figure S9: Joint distribution of CNs and CMs order parameters (OPs) of alkali group and transition metal group entries. Dark color represents high probability.

Table S1: Absorbing species and CNs with only one CM ranking label. Twelve coordinated ($q_{CN-12} \geq 0.2$) entries were excluded as their coordination environments all resemble the cuboctahedral coordination motif, i.e., $q_{cuboctahedral} \geq 0.05$.

Absorbing specie	coordination number	coordination motif ranking label
Si	CN6	*octahedral | pentagonal pyramidal | hexagonal planar*
Al	CN6	*octahedral | pentagonal pyramidal | hexagonal planar*
Cd	CN5	*trigonalbipyramidal | square pyramidal | pentagonal planar*
In	CN6	*octahedral | pentagonal pyramidal | hexagonal planar*
Ge	CN6	*octahedral | pentagonal pyramidal | hexagonal planar*
Ru	CN6	*octahedral | pentagonal pyramidal | hexagonal planar*
Mg	CN7	*pentagonal bipyramidal | hexagonal pyramidal*
Sr	CN4	*tetrahedral | trigonal pyramidal | seesaw like square co-planar*
Mn	CN4	*tetrahedral | trigonal pyramidal | seesaw like square co-planar*
C	CN1	*single bonds*

Table S2: Coordination motif ranking labels prediction accuracy of optimized random forest classifiers on 28 experimental spectra. Although 17 out of 28 spectra have CN-6 *octahedral* | *pentagonal pyramidal* | *hexagonal planar* CM, it does not make the problem easier since those are all test data (not training data) obtained from experiments.

Formula	Space-group	Absorbing species	Correct CN-CM labels	CN-CM in top?	All correct?	Data source
$LiCoO_2$	$R\bar{3}m$	Co	CN-6 *octahedral* | *pentagonal pyramidal* | *hexagonal planar*	Yes	Yes	Ref.[2,3]
$LiNiO_2$	$R\bar{3}m$	Ni	CN-6 *octahedral* | *pentagonal pyramidal* | *hexagonal planar*	Yes	Yes	Ref.[2,3]
NiO	$Fm\bar{3}m$	Ni	CN-6 *octahedral* | *pentagonal pyramidal* | *hexagonal planar*	Yes	Yes	Ref.[2,3]
VO_2	$P2_1/c$	V	CN-6 *octahedral* | *pentagonal pyramidal* | *hexagonal planar*	Yes	Yes	Ref.[2,3]
V_2O_5	$Pmmn$	V	CN-5 *trigonal bipyramidal* | *pentagonal planar* | *square pyramidal*	No	No	Ref.[2,3]
V_2O_3	$R\bar{3}c$	V	CN-6 *octahedral* | *pentagonal pyramidal* | *hexagonal planar*	Yes	No	Ref.[2,3]
$AlPO_4$	$I\bar{4}$	Al	CN-4 *tetrahedral* | *trigonal pyramidal* | *see−saw−like* | *square co−planar*	Yes	Yes	EELS Data Base[4]
B_2O_3	$P3_121$	B	CN-3 *trigonal planar* | *trigonal non−coplanar* | *T−shaped*	Yes	Yes	EELS Data Base[4]
SiO_2	$I\bar{4}2d$	Si	CN-4 *tetrahedral* | *trigonal pyramidal* | *see−saw−like* | *square co−planar*	Yes	Yes	EELS Data Base[4]
Na_2O	$Fm\bar{3}m$	Na	CN-4 *tetrahedral* | *trigonal pyramidal* | *see−saw−like* | *square co−planar*	No	No	EELS Data Base[4]
MnO	$Fm\bar{3}m$	Mn	CN-6 *octahedral* | *pentagonal pyramidal* | *hexagonal planar*	Yes	Yes	XAFS Library[5]
MnO_2	$I4/m$	Mn	CN-6 *octahedral* | *pentagonal pyramidal* | *hexagonal planar*	Yes	Yes	XAFS Library[5]
Mn_3O_4	$I4_1/amd$	Mn	CN-6 *octahedral* | *pentagonal pyramidal* | *hexagonal planar* CN-4 *tetrahedral* | *see−saw−like* | *square co−planar*	Yes	No	XAFS Library[5]
Mn_2O_3	$Pbca$	Mn	CN-6 *octahedral* | *pentagonal pyramidal* | *hexagonal planar*	Yes	Yes	XAFS Library[5]

Continued on next page

Table S2 – *Continued from previous page*

Formula	Space-group	Absorbing species	Correct CN-CM labels	CN-CM in top?	All correct?	Data source
$K_2Cr_2O_7$	$P\bar{1}$	Cr	CN-4 *tetrahedral* | *trigonal pyramidal* | *see−saw−like* | *square co−planar*	Yes	Yes	XAFS Library [5]
K_2CrO_4	$Pnma$	Cr	CN-4 *tetrahedral* | *trigonal pyramidal* | *see−saw−like* | *square co−planar*	Yes	Yes	XAFS Library [5]
Cr_2O_3	$R\bar{3}c$	Cr	CN-6 *octahedral* | *pentagonal pyramidal* | *hexagonal planar*	Yes	Yes	XAFS Library [5]
Na_2CrO_4	$Cmcm$	Cr	CN-4 *tetrahedral* | *trigonal pyramidal* | *see−saw−like* | *square co−planar*	Yes	Yes	XAFS Library [5]
Fe_2O_3	$R\bar{3}c$	Fe	CN-6 *octahedral* | *pentagonal pyramidal* | *hexagonal planar*	Yes	Yes	XAFS Library [5]
FeO	$I4/mmm$	Fe	CN-6 *octahedral* | *pentagonal pyramidal* | *hexagonal planar*	Yes	Yes	XAFS Library [5]
ZnO	$P6_3mc$	Zn	CN-4 *tetrahedral* | *trigonal pyramidal* | *see−saw−like* | *square co−planar*	No	No	XAFS Library [5]
Ni_2O_3	$Cmcm$	Ni	CN-6 *octahedral* | *pentagonal pyramidal* | *hexagonal planar*	Yes	Yes	XAFS Library [5]
CuO	$P4_2/mmc$	Cu	CN-4 *square co−planar* | *rectangular see−saw−like* | *see−saw−like* | *trigonal pyramidal* | *tetrahedral*	No	No	XAFS Library [5]
V_2O_5	$Pmmn$	V	CN-5 *trigonal bipyramidal* | *pentagonal planar* | *square pyramidal*	No	No	XAFS Library [5]
VO_2	$P4_2/mnm$	V	CN-6 *octahedral* | *pentagonal pyramidal* | *hexagonal planar*	Yes	No	XAFS Library [5]
V_2O_3	$Ia3$	V	CN-6 *octahedral* | *pentagonal pyramidal* | *hexagonal planar*	Yes	Yes	XAFS Library [5]
VO	$R\bar{3}m$	V	CN-6 *octahedral* | *pentagonal pyramidal* | *hexagonal planar*	Yes	Yes	XAFS Library [5]
CdO	$Fm\bar{3}m$	Cd	CN-6 *octahedral* | *pentagonal pyramidal* | *hexagonal planar*	Yes	Yes	XAFS Library [5]

References

[1] Mougoyannis, P., (2016). Reactive CaCO3 nucleation and nanoparticles growth in non-aqueous phase. univ. Leeds, School Chem. Proc. Eng. Trans. Rep. 1, 1–10.

[2] Rana, J., Glatthaar, S., Gesswein, H., Sharma, N., Binder, J.R.,

Chernikov, R., Schumacher, G., Banhart, J., (2014). Local Structural Changes in LiMn1.5Ni0.5O4 Spinel Cathode Material for Lithium-Ion Batteries. J. Power Sources 255, 439–449.

[3] Rana, J., Kloepsch, R., Li, J., Scherb, T., Schumacher, G., Winter, M., Banhart, J., (2014). On the Structural Integrity and Electrochemical Activity of a 0.5Li2MnO3·0.5LiCoO2 Cathode Material for Lithium-Ion Batteries. J. Mater. Chem. A 2, 9099.

[4] Ewels, P., Sikora, T., Serin, V., Ewels, C.P., Lajaunie, L., (2016). A Complete Overhaul of the Electron Energy-Loss Spectroscopy and X-Ray Absorption Spectroscopy Database: Eelsdb.Eu. Microsc. Microanal. 22, 717–724.

[5] XAS Spectra Library, https://cars.uchicago.edu/xaslib, accessed Feb 24 2019.
